# Ultra-steep side facets in multi-faceted SiGe/Si(001) Stranski-Krastanow islands

**DOI:** 10.1186/1556-276X-6-70

**Published:** 2011-01-12

**Authors:** Moritz Brehm, Herbert Lichtenberger, Thomas Fromherz, Gunther Springholz

**Affiliations:** 1 Institut für Halbleiter und Festkörperphysik, Universität Linz, 4040 Linz, Austria

## Abstract

For the prototypical Ge/Si(001) system, we show that at high growth temperature a new type of Stranski-Krastanow islands is formed with side facets steeper than {111} and high aspect ratio. Nano-goniometric analysis of the island shapes reveals the presence of six new facet groups in addition to those previously found for dome or barn-shaped islands. Due to the highly multi-faceted island shape and high aspect ratio, the new island types are named "cupola" islands and their steepest {12 5 3} side facet is inclined by 68°to the substrate surface. Assessing the relative stability of the new facets from surface area analysis, we find that their stability is similar to that of {113} and {15 3 23} facets of dome islands. The comparison of the different island shapes shows that they form a hierarchical class of geometrical structures, in which the lower aspect ratio islands of barns, domes and pyramids are directly derived from the cupola islands by successive truncation of the pedestal bases without facet rearrangements. The results underline the key role of surface faceting in the process of island formation, which is as crucial for understanding the island's growth evolution as it is important for device applications.

## Introduction

SiGe islands grown on Si(001) substrates exhibit a large variety of shapes that strongly depend on the Ge growth temperature and thus the thermal energy provided to the system [1]. For very low growth temperatures of *T*_*Ge *_<400°C, atom surface diffusivity is low, *i.e*., Ge atoms are incorporated before they can form three-dimensional (3D) island clusters and the elastic energy stored in the 2D film is relaxed by misfit dislocation formation [2]. For temperatures between 400°C and 550°C, rectangular {105}-faceted islands called "hut clusters" [3] form. Their elongated rectangular base is caused by kinetic barriers at the island edges [4] and their aspect ratio (AR), defined as ratio of height versus square root of the island's base area, is <0.1. For higher growth temperatures in the range of 600°C to 720°C, the islands evolve into square-based {105} faceted pyramids (P) [3] with AR = 0.1 and, to multi-faceted domes (D) [5] with AR ≈ 0.2. The steepest side facets of the domes are {15 3 23} facets that are inclined at an angle of 33.6° with respect to the (001) substrate surface, while the inclination angle for the {105} pyramid facets is only 11.3°. The pyramids and domes form a bimodal [5,6] or uniform monomodal [7] island size distribution depending on the Ge coverage and growth conditions. At high coverage, plastic relaxation sets in and dislocated islands denoted by superdomes (SD) [8] form. These usually have similar side facets and aspect ratios as the coherent dome islands, but have a significantly larger size. For even higher growth temperatures of 720°C to 800°C, barn-shaped islands (B) [9] are formed along with domes and pyramids. These barns exhibit all facets of the domes, as well as additional steeper ones like the {111} facets, which are inclined by 54.7° to (001). The aspect ratio of the barns is therefore increased to around AR ≈ 0.3 [10]. This indicates a general trend that at higher temperatures islands with steeper facets and thus higher aspect ratio are formed. Islands with larger volume are also expected to nucleate when the growth rate is lowered or a SiGe alloy is deposited. Although the {111} barn side facets were suggested to be the steepest facets under the usual epitaxial conditions [11], it seems natural to go beyond the previously employed temperature regimes to see if this trend to higher aspect ratios and steeper side facets can be extended.

In this study, Ge islands were grown on Si (001) at conditions very close to thermal equilibrium at *T*_Ge _= 900°C. Using advanced image processing for nano-goniometric analysis of atomic force microscopy (AFM) images, it is demonstrated that under these conditions islands with ultra-steep side facets inclined by up to 68° to the (001) substrate surface are formed. As derived from detailed surface orientation maps, six new facet groups with relatively low miller indices are identified, of which the {12 5 3} facets are found to be the steepest ones. Comparison of the facet areas allows assessing their relative stability, showing that the {12 5 3} and {715} facets are found to be the most stable ones of the new facet classes. The new SiGe islands exhibit an aspect ratio as high as 0.384 and their shape approaches that of a multi-faceted half sphere. Thus, these new islands are referred to as "cupola" islands to distinguish them from the previously observed island shapes formed at lower growth temperatures. Shapes of SiGe islands have been traditionally derived by referring to architectural shapes. While the terms "hut-cluster" and "pyramids" describe the experimental island shapes rather well, in the case of "domes" the experimental island shapes are far from being half spheres with an aspect ratio of only 0.2 compared to 0.56 for a half sphere. The same applies to the "barns", which experimentally exhibit a fourfold in-plane symmetry, whereas a barn has only a two fold symmetry axis.

## Experimental

The samples were grown by solid source molecular beam epitaxy (MBE) on high-resistivity 4" Si (001) wafers. After *in-situ *oxide desorption at 950°C for 20 min, a 45 nm-thick Si buffer layer was grown at substrate temperatures ramped up from 550 to 700°C [6,7]. Thereafter, 8 ML of Ge were deposited at a rate of *R*_Ge _= 0.05 Å/s and a growth temperature of *T*_Ge _= 900°C. The samples were *ex-situ *characterized by a Digital Instruments Dimension 3100 AFM in tapping mode using Olympus cantilevers with sharpened Si tips with half opening angle of 15° and nominal tip radius of 2 nm. For sufficiently large islands, therefore, facet angles as steep as 75° can be measured without detrimental influence of the tip geometry. Advanced image processing and correction software was used for generation of distortion-free AFM images and surface orientation maps, as well as for statistical evaluation of the facet areas of larger ensembles of Ge islands. This allows precise nano-goniometry of all facet angles and the precise identification of their {*hkl*} indices as well as the assessment of their relative stability.

## Results and discussion

**Figure 1:**
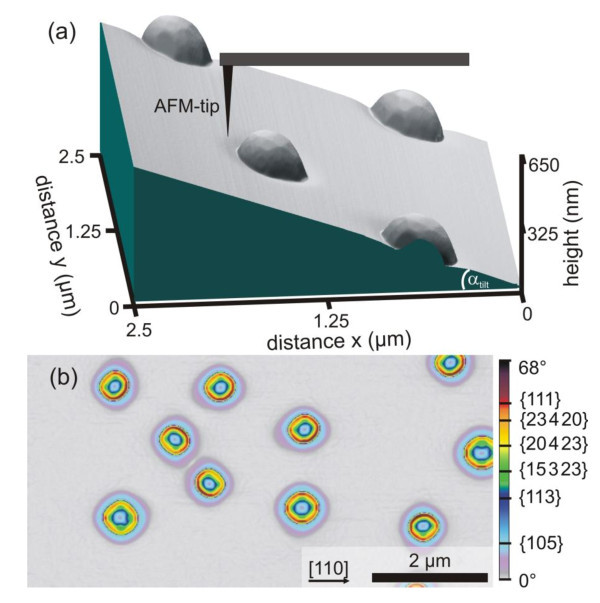
**AFM images of the cupola islands**. **(a) **3D AFM micrograph (2.5 × 2.5 μm^2^) of SiGe islands measured after sample mounting on a wedge shape sample holder with tilt angle α_tilt _= 14°. **(b) **Larger scale (8 × 4 μm^2^) AFM micro-graph of the non-tilted sample for comparison, where the color scale represents the local surface slope with respect to the (001) surface in the range from 0° to 68° (surface angle image), as indicated by the color bar on the right hand side, where the surface slopes of the characteristic dome facets are indicated. Evidently, most of the islands exhibit very steep side facets at the perimeter that are inclined by up to 68° (red color) with respect to the (001) surface normal.

At 900°C and 8 ML Ge coverage, islands with a density of 2.4 × 10^7 ^cm^-2 ^and a typical height of 156 nm and diameter of 400 nm are formed with a very high uniformity and narrow size dispersion of only ± 0.8%, as obtained from statistical analysis of large-scale AFM images. The islands are significantly larger than the barns and domes obtained at lower *T*_Ge_, where for 700°C typical values of 20 and 120 nm for height and diameter are found [6] and the density of approximately 10^9 ^cm^-2 ^is about a factor of 50 higher than that found for *T*_Ge _= 900°C. The higher growth temperature also results in a higher degree of Si/Ge intermixing, as estimated from the ratio of 5 ML Ge incorporated in the dots (8 ML minus 3 ML assumed to be in the wetting layer) over the total island volumes measured by AFM. This yields an average Ge content of *x*_Ge _= 20% within the islands (not accounting for possible Ge desorption) compared to typically *x*_Ge _= 40% for the domes grown at 700°C [1,6]. The low Ge content is also the reason that the islands are still dislocation free in spite of their larger size. This strong intermixing is also indicated by about 30 nm deep trenches formed around each island, which are visible as light blue and violet rings in the AFM images of Figure 1b, d. Such trenches are also present for dome and barn islands [1], but their depth is significantly smaller than in our case.

**Figure 2:**
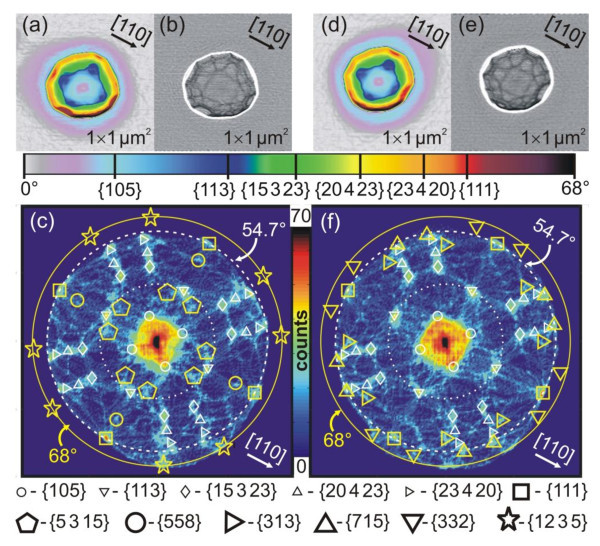
**Surface angle images, laplacian images and surface orientation maps for two selected cupola islands**. Surface angle images (a, d) and respective Laplacian images (b, e) with pure edge contrast of two cupola-shaped SiGe islands obtained by AFM. The calculated surface orientation maps of the two islands are shown in panels **(c) **and **(f)**, in which the bright spots labeled by the different symbols indicate all facets of the islands. The large bold symbols mark the five new facets groups and the {111} facets, the other facets also observed for barn or dome-shaped islands are marked by the small white symbols as listed below panels (c) and (f). For reasons of visibility not all the facets are marked with symbols in both images. The dotted, dashed and solid circles in the SOMs mark all surfaces inclined by *θ *= 25.3°, respectively, 54.7° and 68° to the (001) substrate plane indicate the locations of the {113}, {111} and {12 3 5} facet spots, respectively, where the latter are the steepest side facets of the islands. The averaged tilt and azimuth angles θ and φ of all facets are listed in Table 1.

To obtain detailed information on the island shapes, high-resolution AFM images were recorded on a magnified scale as shown in Figures 1 and 2. In general, the fidelity of AFM imaging of surface nano-islands decreases with increasing slope of the side wall facets due to the limitations in feedback gain and distortions caused by the tip-sample convolution. Moreover, the projected facet areas "seen" by the AFM tip, *i.e*., facet areas projected to the image plane becomes smaller with increasing facet slope. Thus, even though a steep facet might have a large *true *area, it will be still difficult to measure its size and inclination by AFM. A straightforward method to increase the projected side facet areas is to perform AFM measurements on samples mounted on tilted wedge-shaped sample holders, as shown schematically in Figure 1a. The maximum allowable tilt angle, however, is generally limited by the vertical AFM scan range as well as the overall tip/cantilever geometry. In the present work, the samples were glued on a wedge providing a tilt of α_tilt _≈ 14° and a corresponding representative 3D AFM topography is displayed in Figure 1a. In this way, the angular resolution on the upper side of the islands can be notably increased.

Figure 1b shows a large-scale (8 × 4 μm^2^) micrograph of the non-tilted sample for comparison, where the color scale represents the local surface slope on the sample with respect to the (001) substrate. We will refer to this kind of representation as surface angle image (SAI). The color scale on a scale from 0° to 68° was chosen such that each known SiGe island facets from {105}, {113}, {15 3 23}, {20 4 23}, {23 4 20} to {111} [3,5,9,11,12] correspond to one color as indicated on the scale bar on the right-hand side. This reveals the exact location of the respective facets on the islands in the SAI image [7, Hackl F, Grydlik M, Brehm M, Groiss H, Schäffler F, Fromherz T, Bauer G, Microphotoluminescence and perfect ordering of SiGe islands on pit-patterned Si(001) substrates, submitted]. From the color coding, it is evident that essentially all islands exhibit side facets significantly steeper than the 54.7° inclined {111} facets. These steep facets shown in Figure 1b correspond to the dark red and black areas at the perimeter of the islands. Additional evidence for the existence of steeper island facets comes from evaluation of the island aspect ratios obtained from the measured island heights and the square root of the base areas. We find an aspect ratio as large as AR = 0.384, which significantly exceeds that of dome or barn islands (AR ≈ 0.2 [5] and ≈0.3 [9], respectively). Thus, it is evident that the surface of our islands contains steeper side facets than usually observed in SiGe islands.

For detailed facet analysis, zoomed-in AFM images of a large number of individual islands were recorded using the wedge-shape sample holder. The result is exemplified in Figure 2 for two SiGe islands, where (a) and (d) represent the SAI images with slope contrast similar as to that in Figure 1, and 1(b) and 1(e) their Laplacian transformation, in which the grey-scale corresponds to the local surface curvature in the image, thus producing a strong edge contrast between the facet areas of the islands [5]. This image representation clearly reveals that a large number of side facets with different inclination and azimuth angles are present on the islands and that the side wall inclination at the island edges is larger than 60°. For these reasons, we call these islands "cupola" islands to distinguish them from the pyramids, domes and barns observed in previous studies [3,5,9].

**Figure 3:**
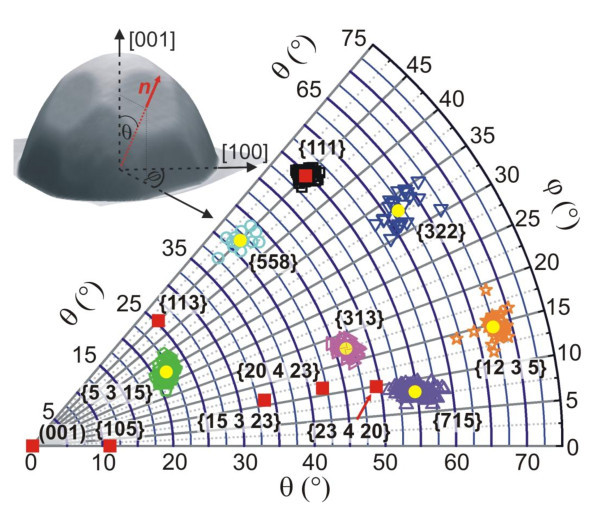
**Unit stereographic sector showing by the open symbols the measured angles (*θ*, *φ*) of all new facets of 30 individual half-sphere SiGe islands on Si(001) substrate grown at 900°C as derived from the surface orientation maps exemplified in Figure 2**. Full yellow circles mark the average (*θ*, *φ*) values for each facet group. The previously reported dome and barn facets of {105}, {113}, {15 3 23} and {111} are indicated by the red squares. The AFM insert at the top illustrates the stereographic projection of the normal vector and its relation to the (θ, *φ*) angles for the case of a SiGe dome island.

For nano-goniometric analysis, the AFM images recorded on the wedged tilted sample holder were first numerically rotated such that the substrate surfaces lay within the image plane. Then, these images were additionally rotated around the island's z-axis so that the [100] directions became aligned parallel to a fixed axis in the image plane. This image transformation was performed employing rotational matrices and using the 2D surface between the islands as well as the known {111} island facets as references. To precisely determine all facet orientations from such transformed AFM images, surface orientation maps (SOM, sometimes also called facet density plots [12,13]), were generated. These are obtained by calculating for each image point the surface normal vector [*hkl*] using the nearest-neighboring image points to define the local surface plane. The intersection points of the surface normal vectors and the half-sphere seen from top as projection yields the 2D polar coordinates (*θ*, *φ*) of the [*hkl*] vectors, where *θ *is the inclination angle between [*hkl*] and the [001] substrate normal and *φ *denotes the in-plane azimuth angle of the [*hkl*] vector with respect to the [100] substrate direction as illustrated by the 3D AFM insert of Figure 3. The intensity of each point (*θ*, *φ*) in the surface orientation map represents the relative abundance of surface points with a given local [*hkl*] orientation, thus representing a 2D histogram of surface orientations in the AFM image. This procedure was first successfully applied by Lutz et al. [13] for characterization of SiGe layer morphologies and later by others for Stranski-Krastanow islands [12] and other surface undulations [14].

For the two islands depicted in Figure 2, the surface orientation maps obtained from the AFM images are shown in Figure 2c, f, respectively. Clearly, a large number of more than 50 facet spots appear in these SOMs, where each facet class is labeled by a different symbol as listed in the lower part of Figure 2. The dotted and dashed circles indicate the surfaces with inclination of *θ *= 25.2° and 54.7°, which include the prominent {113} and {111} facets observed for dome and barn islands, respectively. The facet spots outside of the dashed circle correspond to facets steeper than {111} and are indicated by open star symbols in Figure 2. Their inclination is found to be approximately *θ *= 68° as marked by the outer solid circle in the SOMs and they are located at the perimeter of the islands (dark red and black areas in the surface angle AFM images of Figure 2a, d). In the SOMs, the location of the known SiGe facets of domes and barns are represented by small white symbols, i.e., circles for {105}, differently oriented triangles for {113}, {20 4 23} and {23 4 20}, diamonds for {15 3 23} and squares for {111} facets, which are all also present in the surface orientation maps of our cupola islands. Facets with inclinations lower than *θ *<11° are not well resolved due to the broad back-ground contribution from the rounded 30 nm deep trenches around the islands, visible as light blue and violet rings around the islands in the SAI AFM images of Figure 2a, d. Apart from these known island facets, a large number of additional facet spots are observed in the SOMs as marked by the bold symbols in Figure 2.

For identification and indexing of the new island facets, the surface orientation maps of more than 30 individual islands were analyzed. The results are compiled in Figure 3, where the measured values of *θ*, *φ *of all new SOM facet spots of the islands are plotted on top of each other. Evidently, the measured facet angles cluster around certain *θ*, *φ *values, indicating that they correspond to well-defined {*hkl*} surface orientation. The average *θ*, *φ *values computed for each group of data points are given in Table 1 (*θ*_exp_, *φ*_exp_), where the spread, *i.e*., standard deviation is represented by the error values. For assignment of miller indices, the measured angles were then compared to the theoretical inclination and azimuth angles *θ*_th_, *φ*_th _of a large number of {*hkl*} surfaces, and the ones with best match and reasonable low miller indices are listed in Table 1. In this way, six new facet classes of the SiGe islands were identified, namely, {5 3 15}, {558}, {313}, {715}, {322} and {12 3 5} facets in the order of increasing inclination angle, where the {12 3 5} facets represent those with the steepest inclination angle of 68°. As proved in Table 1, for these facets, the theoretical (*θ*, *φ*) values are within the experimental error bars or at least within 1° of the experimental values. The facet positions of the new {*hkl*} facets are marked by the bold symbols in the SOMs of Figure 2, where pentagons indicate {5 3 15}, circles {558}, triangles with different orientation {313}, {715} and {322} and stars the steepest {12 3 5} facets.

**Table 1:** Results of the facet analysis for SiGe island formed at high temperatures, listing all experimentally observed {*hkl*} island facets with the corresponding experimental and theoretical values *θ *and *φ *as well as the facet area relative to {111}.

Facet	***θ***_**th **_**(°)**	***θ***_**exp **_**(°)**	***φ***_**th **_**(°)**	***φ***_**exp **_**(°)**	Also occurs for	Facet area relative to {111} (%)
{105}					P, D, B	
{5 3 15}	21.2	21.8 ± 0.1	30.1	29.1 ± 0.3	*	21 ± 1
{113}	25.2	25.1 ± 0.1	45.0	45.0 ± 0.2	D, B	77 ± 4
{15 3 23}	33.6	33.9 ± 0.1	11.3	11.6 ± 0.1	D, B	44 ± 2
{558}	41.5	41.6 ± 0.5	45.0	44.9 ± 0.4	*	19 ± 1
{20 4 23}	41.7	40.7 ± 0.1	11.3	11.6 ± 0.2	B	35 ± 2
{313}	46.5	46.6 ± 0.2	18.4	17.4 ± 0.2	*	29 ± 1
{23 4 20}	49.4	49.3 ± 0.2	9.9	10.4 ± 0.2	B	23 ± 12
{715}	54.7	54.7 ± 0.2	8.1	8.2 ± 0.1	*	62 ± 3
{111}	54.7	54.7 ± 0.1	45.0	45.0 ± 0.1	B	100 ± 7
{322}	61.0	61.7 ± 0.6	33.7	33.0 ± 0.3	*	32 ± 2
{12 3 5}	68.0	67.4 ± 0.4	14.0	14.7 ± 0.2	*	53 ± 2

* New facets

In Table 1 the {*hkl*} facets are ordered from top to bottom according to increasing inclination angle *θ*. The fifth column indicates for which other island geometries the respective facets also occur, with P = pyramids [3], D = domes [5], B = barns [9]. Stars indicate the facets that are only observed for our cupola islands and have not been reported before. The last column gives the relative surface area of each facet class with respect to the {111} facets of the cupola islands. All experimental values represent the results from averaging over the facet analysis of 30 individual islands measured by AFM. The standard deviation of each value from the average is represented as error value. For the given (*hkl*) assignment, the theoretical and experimental angles agree within less than 1°.

Evidently, with this assignment all facet spots in the SOMs are well reproduced. To our knowledge, only the existence of a stable {313} facet was previously reported for highly miscut Si and Ge surfaces [15]. It is noted, that in the facet assignment, we have selected {*hkl*} indices with the smallest possible value for (*h*^2 ^+ *k*^2 ^+ *l*^2^). Obviously, there are also other surface orientations with higher {*hkl*} facet indices with theoretical angles *θ *and *φ *close to the experimental values like {7 4 20}, {9 5 26} or {16 9 46} instead of {5 3 15} facet; {13 4 13}, {16 5 16}, {19 6 19}, {22 7 22} or {23 8 23} instead of {313}; {20 13 13} for {322} and {19 5 8} for {12 3 5}, but were not considered for further analysis.

To assess the relative stability of the various facets of the islands, we have determined precisely the unprojected surface area for each surface orientation. In addition, the resulting values were averaged over all 30 islands used for the analysis. The average facet areas of each {*hkl*} class were then normalized to that of the largest island facets, which are the {111} side facets. The results are listed in the last column of Table 1. We take the surface area as a qualitative measure of the facet stability, although the situation is different as compared to the Wulff construction of bulk crystal shapes because the island shape in our case also depends on the elastic lattice mismatch strain imposed by the Si substrate. According to Table 1, with 100%, respectively, 77% the {111} and {113} facets have the largest surface areas. This agrees well with the fact that these surfaces are well-known thermodynamically stable surfaces of bulk Si and Ge [16]. Also, the newly found steep {715} and {12 3 5} facets with two facets per 45° unit sector, each area counted separately, seem to be surprisingly stable since their average areas (61.6 and 53%) are more than half of the {111}-facet area. In comparison, the area of the {15 3 23} facets prominent for dome islands is only 43.6% of the average {111}-facet area size. This indicates a quite high stability of the new {715} and {12 3 5} facets. Facets with lower areal coverage of around 30% of the {111} facets are the {20 4 23}, {313} and {322} facets, and the {23 4 20}, {5 3 15} and {558} facets exhibit relative facet area ratios of only about 20%.

**Figure 4:**
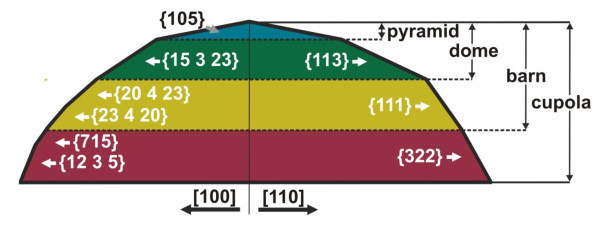
**Cross section of the cupola-shaped SiGe islands along the [100] and [110] substrate direction (left and right hand side, respectively)**. As indicated by the horizontal lines, from this shape the other known SiGe island shapes of barns, domes and pyramids can be directly derived.

Our results clearly corroborate the general trend that with increasing growth temperature and island volume, the aspect ratio of SiGe islands, *i.e*., height versus average base diameter, successively increases stepwise from AR = 0.1 to 0.2, 0.3 and finally 0.384, when going from pyramids, to domes, barns and finally cupola islands. The aspect ratio of 0.384 is still less than the reported AR for inverted {111}-pyramids grown in {111}-pits [17] and less than for truncated {111}-pyramids grown by liquid phase epitaxy [18]. In addition, the number of side facets and their maximum inclination angles also increases with increasing aspect ratio. Concerning the whole group of SiGe islands, a remarkable hierarchy of shapes emerges, in which steeper islands are always derived from shallower islands by adding successively steeper pedestal elements to the base of the islands. This is demonstrated schematically in Figure 4 by the illustration of the cupola island cross-section. As indicated by the dashed horizontal lines, the other SiGe island shapes of pyramids, domes and barns can be simply derived by cutting the cupola islands at different height levels corresponding to the given aspect ratios of the different island types. Going with these plane cuts from the top to the bottom, we can recover each known SiGe island shape of pyramids, domes and barns from the cupola islands without further facet rearrangements. The formation of higher aspect ratio island shapes can thus be considered as adding a steeper faceted base in each transition to the next hierarchical level of island shapes without changing the existing upper part, which obviously represents a very natural way of transformation of the flatter pyramids and domes to the almost hemispherical final cupola islands.

## Conclusions

In conclusion, six new facet groups of SiGe Stranski-Krastanow islands on Si (001) grown at high substrate temperature were identified by AFM-based nano-goniometry and facet analysis. These islands exhibit a significantly higher aspect ratio of 0.384 compared to less than 0.3 for the known pyramid, dome and barn-shaped islands and they comply with the general trend of increasing aspect ratio with increasing growth temperature. Due to the highly multi-faceted island shape and high aspect ratio, the new island types are named "cupola" islands. Their steepest side facet with {12 5 3} orientation is inclined by 68° to the Si substrate surface, which is significantly larger than the inclination of the {111} side facets that until now were considered as the steepest SiGe island facets. From the analysis of the facet areas, the relative stabilities of the new facet groups were assessed and it was found that their stability was similar to that of the common {113} and {15 3 23} facets of the dome islands. Moreover, the comparison of the different Stranski-Krastanow island shapes shows that they form a hierarchical class of geometrical structures, in which the lower aspect ratio islands of barns, domes and pyramids are directly derived from the cupola islands by successive truncation of the pedestal bases without facet rearrangements. In all, the presented results underline the key role of surface faceting in the process of island formation, which is crucial to understanding their growth evolution as is important for device applications.

## Abbreviations

3D: three-dimensional; SOM: surface orientation maps; AFM: atomic force microscopy; MBE: molecular beam epitaxy; SAI: surface angle image.

## Competing interests

The authors declare that they have no competing interests.

## Authors' contributions

MB designed and carried out the experiments and the statistical analysis, HL designed the study of the SOM analysis, MB, TF and GS participated in the manuscript design and coordination. All authors read an approved the final manuscript.
